# Home Department

**Published:** 1886-11

**Authors:** 


### HOME DEPARTMENT.

**Bananas and Cream.**—Peel, slice and place in a dessert dish; serve raw with powered sugar and cream.

**Lemon Tea.**—Place in each cup a lump or two of sugar, a slice of lemon and a little extra juice, and some small pieces of ice; fill up the cup with cold tea. Ice can be broken in small pieces by placing a lump in a piece of coarse, strong cloth, and pounding it with a mallet.

**Pepper Sauce Relish.**—Chop onions fine, let soak half an hour in weak salt water; put in a strainer cloth and squeeze dry; to each teacupful of onion add a heaping teaspoonful of white pepper (black is as good but don't look well) and mix, put in a pickle well and cover with vinegar.

**Milk Custard.**—Soak a bit of rennet over night in tablespoonful of water. In the morning, warm as much milk as you will need, pour it in an earthern dish, add the rennet water and a pinch of salt, stir the milk well, let it stand until it thickens, then set in as cold a place as you have. Eat sugar and milk or cream on it, using any flavoring yŏu like.

**Refreshing Drink.**—Pour one quart of boiling water on one and one-half pounds of sugar, one-half pint of molasses, two ounces of tartaric acid and half an ounce of essence of sassafras. When cold, bottle and cork tight and keep in a cool place. When wanted to use, take three teaspoonfuls of this syrup in three-fourths of a tumbler of water, put in one-fourth of a teaspoonful of soda, stir and drink immedia.tely

**To Preserve Eggs.**—According to *Mittheilungen Landwirthschaft*, vaseline is a good preservative for eggs. The eggs should be thoroughly washed and rubbed with vaseline previously melted with three-tenths per cent. of salicylic acid. The operation should be performed twice, the latter one month after the former. On boiling the skin of vaseline easily separates from the eggs. Eggs thus treated are said to keep perfectly fresh for a year.

**Coffee and Egg for Sick Persons.**—A sick person wanting nourishment and having lost appetite, can often be sustained by the following, when nothing else could be taken: Make a strong cup of coffee, adding boiling milk as usual, only sweetening rather more; take an egg, beat yolk and white together thoroughly; boil the coffee, milk and sugar together, and pour it over the beaten egg in the cup you are going to serve it in. This simple receipt is used frequently in hospital practice·

**Vegetable Salads.**—The markets at this season of the year afford such an unlimited variety of vegetbles that with a little attention to the different modes of preparing them every housekeeper will be enabled to provide numerous and agreeable changes for her table, as well as contribute largely to the health of the family, for it is impossible to overestimate the importance of vegetables as daily articles of diet. Vegetable salads are among the most agreeable and wholesome dishes that can be prepared for a light summer repast; besides being very economical, they are easily made, tempt the appetite, and impart a flavor to the rest of the meal. If desired in perfection, care should be taken in their preparation. The dressing for a variety of light salads is the same but different flavors can be delicately added, and skill will soon be acquired by practice if due attention is paid to the directions given. In making salads the freshest olive oil should be used.

**Milk Broths** are the best of food for young growing bairns. Milk broth proper is made of pearl barley and new or skimmed milk, with sugar to taste. This is the way: A piece of fresh butter the size of a walnut is put into the broth pan and allowed to melt, then the pan is turned about, so that the oiled butter will run all over the bottom. This is done as a preventive against the barley sticking to the bottom. A quarter of a pound of washed pearl barley is put in with three quarts of milk. This is placed on a gentle fire and allowed to boil. It is now drawn to the side and boiled very gently for three hours, being stirred occasionally to prevent the barley from sticking to the bottom, getting "singed" or "sung," in the event of which the broth is spoiled. Before serving, sugar to taste is added. When done, the milk will be a thick yellowish creamy liquid. It is served in soup plates, with bread or oatcakes.

**The Diet of Strong Men.**—The Roman soldiers, who built such wonderful roads and carried a weight of armor and luggage that would crush the average farm hand, lived on coarse brown bread and sour wine. They were temperate in diet, and regular and constant in exercise. The Spanish peasant works every day and dances half the night, yet eats only his black bread, onion and wàtermelon. The Smyrna porter eates only a little fruit and some olives, yet he walks off with his load of 800 pounds. The coolie, fed on rice, is more active and can endure more than the negro fed on fat meat. The heavy work of the world is not done by men who eat the greatest quantity. Moderation in diet seems to be the prerequisite of endurance.

